# A142 ENDOSCOPIC ULTRASOUND-GUIDED GASTROENTEROSTOMY: A CASE SERIES AND REVIEW OF LITERATURE

**DOI:** 10.1093/jcag/gwae059.142

**Published:** 2025-02-10

**Authors:** C Shamatutu, R Trasolini

**Affiliations:** Medicine, The University of British Columbia, Vancouver, BC, Canada; Medicine, The University of British Columbia, Vancouver, BC, Canada

## Abstract

**Background:**

Gastric outlet obstruction (GOO) refers to the clinical syndrome of epigastric pain and postprandial vomiting due to mechanical obstruction in the distal stomach or proximal duodenum. Management is undertaken to alleviate symptoms with the aim of maintaining nutritional status and improving quality of life. Endoscopic gastroduodenal stenting and surgical gastroenterostomy (SGE) are two established methods in the management of GOO. Enteral stenting is associated with stent malfunction such as migration and obstruction often requiring reintervention; SGE is associated with a higher degree of morbidity and mortality compared to endoscopic approaches. Endoscopic ultrasound-guided gastroenterostomy (EUS-GE), with the placement of a lumen-apposing metallic stents (LAMS) from the stomach to the small bowel distal to the obstruction, has emerged as an alternative approach in the management of GOO.

**Aims:**

To describes a case series of patients who underwent EUS-GE and review the relevant literature.

**Methods:**

This was a single-centre retrospective case series of 9 patients with GOO who underwent EUS-GE at Vancouver General Hospital. We looked at technical success, clinical success, and adverse outcomes. Technical success was defined as ability to place a LAMS between the stomach and the small bowel. Clinical success was defined as the patient’s ability to tolerate oral intake.

**Results:**

A total of 9 patients with GOO underwent EUS-GE. The median age was 65. 5 were females (55%) and 4 (45%) were males. GOO was due to malignant tumors or metastases in 8 cases (89%) and in one case was due to afferent limb syndrome after pancreaticoduodenectomy. The technical and clinical success rate was 100%. The stents had a diameter of 15 mm in 7 cases (77%). The stents had a saddle length of 10mm in 7 cases (77%). All cases were performed under general anesthesia. There were no adverse outcomes. The median time to discharge was 8 days. At the time of the study, 5 patients had deceased, with a mean survival of 17 weeks.

We identified two systematic reviews and meta-analyses looking at EUS-GE. The majority of data comes from retrospective studies. They assessed 285 and 260 patients. Technical success was achieved in 92-93.5% of cases, clinical success was achieved in 90% across both studies, adverse outcomes occurred in 12% of cases in both studies.

**Conclusions:**

EUS-GE is an alternative endoscopic approach in the management of GOO with the ability to ameliorate symptoms of GOO and improve quality of life. It is less invasive than SGE and provides a more durable approach compared to endoscopic stenting. We describe a series of patients at VGH who underwent EUS-GE with a technical and clinical success rate similar to that described in the literature. Further randomized studies comparing the different modalities of treatment for GOO are needed.

**Funding Agencies:**

None

